# Genetic associations of the thyroid stimulating hormone receptor gene with Graves diseases and Graves ophthalmopathy: A meta-analysis

**DOI:** 10.1038/srep30356

**Published:** 2016-07-26

**Authors:** Haibo Xiong, Mingxing Wu, Hong Yi, Xiuqing Wang, Qian Wang, Sophia Nadirshina, Xiyuan Zhou, Xueqin Liu

**Affiliations:** 1 Department of Ophthalmology, the Second Affiliated Hospital of Chongqing Medical University, Chongqing, China; 2 Department of Ophthalmology, Chongqing General Hospital, Chongqing, China; 3 Centre for Nuclear Receptors and Cell Signalling, Department of Biology and Biochemistry, University of Houston, Houston, TX, USA

## Abstract

Graves’ disease (GD) is a common thyroid disease, and Graves ophthalmopathy(GO) is the most common extra-thyroidal manifestation of GD. Genetic associations of the thyroid stimulating hormone receptor (TSHR) gene with GD and GO have been studied in different population groups for a long time. We aimed to obtain a more precise estimation of the effects of TSHR single nucleotide polymorphisms (SNPs) on GD/GO using a meta-analysis. Publications were searched on Pub Med and EMBASE up to December 30, 2015. Eight studies involving three SNPs (rs179247, rs12101255, and rs2268458), which included 4790 cases and 5350 controls, met the selection criteria. The pooled odds ratios (OR) and the 95% confidence intervals (CI) were estimated. SNPs rs179247 (dominant model [GG + GA vs. AA]: OR = 0.66, 95%CI: 0.61–0.73, P = 0.000, I^2^ = 0%) and rs12101255 (dominant model [TT + TC vs. CC]: OR = 1.67, 95%CI: 1.53–1.83, P = 0.000, I^2^ = 0%) were significantly associated with GD in all of the genetic models. TSHR rs12101255 and rs2268458 polymorphisms had no association between GO and GD (GD without GO). The results indicate that rs179247 and rs12101255 are likely to be genetic biomarkers for GD. Further studies with different population groups and larger sample sizes are needed to confirm the genetic associations of the TSHR gene with GD/GO.

Graves’ disease (GD) is the most important factor of hyperthyroidism and is a typical autoimmune thyroid disease^1^. Approximately 3% of women and 0.5% of men all over the world suffer from this disease^2^. GD is characterized by a high level of thyroid hormone, a diffuse goitre, a positive test for thyroid stimulating hormone receptor antibody (TRAb), Graves ophthalmopathy and anterior tibia mucous oedema. Approximately 25–50% of GD patients have a clinical manifestation of Graves ophthalmopathy (GO), which is also called thyroid-associated ophthalmopathy (TAO)^3^,^4^. As the most common clinical manifestation of GD, GO is characterized by the retraction of the upper eyelids, chemosis, palpebral oedema, exophthalmus and extra ocular muscle hypertrophy.

Thyroid stimulating hormone receptor (TSHR) is expressed by the plasma membrane of thyroid follicular cells (TFC)^5^. THSR has a central role in the course of the disease and is the main auto antigen in GD^6^. Auto antibodies for TSHR result in GD with over-activity of the thyroid gland^7^. Thyroid follicles synthesize the TSHR glycoprotein, which activates orbital fibroblasts^8^. This activation leads to a pathological change in surrounding tissue and cells^9^.

The pathogenesis of GD/GO is complex and multifactorial with several environmental and genetic factors. Because TSHR is the main auto antigen in GD and because the titre and the activity of the TSHR autoantibody are positively associated with the severity of GO^10^, the TSHR gene has been studied as a central susceptibility gene for both GD and GO^6^. The TSHR gene is considered to be the main gene that contributes to GD^11^. TSHR gene polymorphisms may influence the protein structure, which alter the autoimmune response against the TSHR in GD and GO^12^.

Genetic associations of immunoregulation and thyroid-specific genes with GD/GO have been studied in different people for a long time. At first, three SNPs (rs61747482, rs2234919 and rs1991517) were studied. However, they were finally excluded because of their frequent presence in healthy people^13^. Later, TSHR gene polymorphisms associated with GD/GO susceptibility were found to be located in intron 1. Three SNPs (rs179247, rs12101255 and rs2268458) were found to be strongly associated with GD/GO. Additionally, two TSHR SNPs on chromosome 14q31 had been found in a genome-wide association study; these SNPs were closely associated with GD^14^,^15^. Though the genetic associations of the TSHR gene have been studied in different ethnic groups for a long time, there is still controversy about the genetic associations for both GD^16^,^17^ and GO^18^. Therefore, we conducted a meta-analysis to investigate the genetic associations of TSHR gene polymorphisms with GD/GO.

## Results

### Characteristics of the articles in the analysis

**Figure 1:**
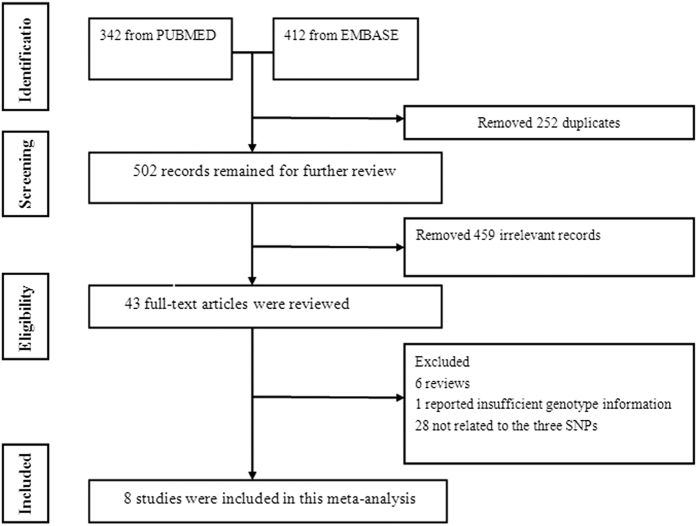
Flowchart of the study selection. SNPs: single nucleotide polymorphisms.

First, 754 articles were selected from the databases, and 252 duplicate articles were removed. Among the remaining articles, 459 were not related to the topic. A total of 43 relevant articles were identified for further study. Of those, 35 articles did not meet the inclusion criteria, including 6 reviews. Additionally, 1 study did not have sufficient data, and 28 studies did not study the three SNPs. In the end, we selected 8 studies that met the inclusion criteria, including 5 studies that involved 4821 GD patients and 4846 healthy controls^12^,^19^,^20^,^21^,^22^ and 5 studies that involved 904 GO patients and 924 GD patients^20^,^22^,^23^,^24^,^25^. Importantly, the study by Ploski included 3 different groups of cases and controls^12^. These studies included European (n = 5), Asian (n = 2), and Latin American (n = 1) groups. All articles stated the GD and GO diagnostic criteria. Five studies included the Hardy-Weinberg equilibrium (HWE) results. The HWE for the other 3 studies was tested by our investigators^21^,^24^,^25^. All of the studies were in HWE. One study tested a non-genetic risk factor (smoking)^23^. All of the studies matched in age distribution, and no adjustment to the factors was reported. Figure 1 illustrates the flow chart of the study selection. Tables 1 and 2 illustrate the primary characteristics of the articles that met the inclusion criteria.

### Genetic associations of the TSHR polymorphism with GD/GO

**Figure 2:**
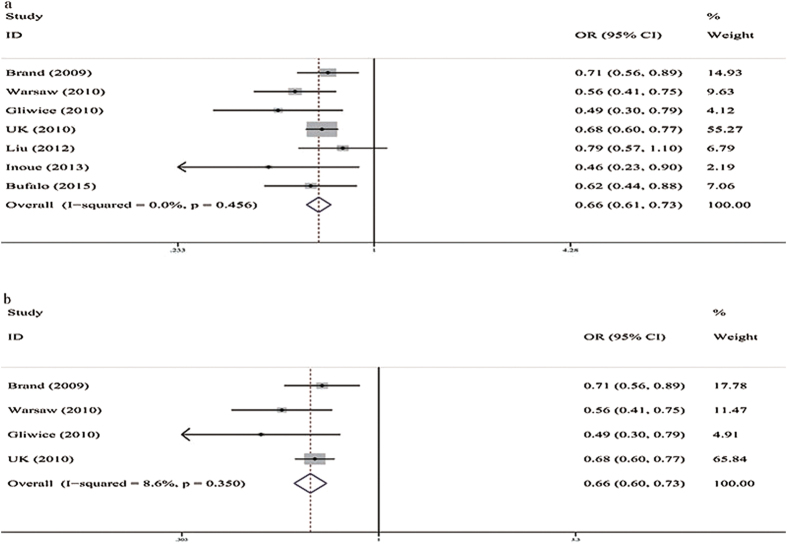
Forest plots for associations between TSHR rs179247 A/G SNP and GD in the dominant genetic model (GG + GA vs. AA). (**a**) Total population; (**b**) Caucasian group.

**Figure 3:**
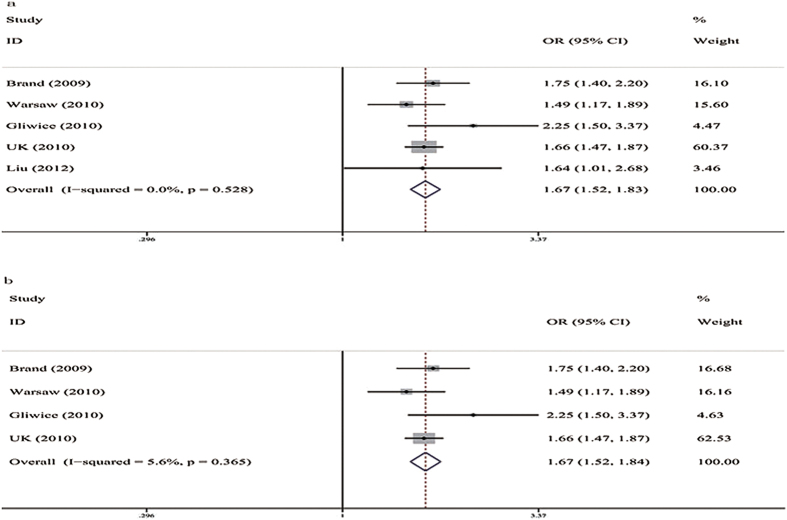
Forest plots for associations between TSHR rs12101255 C/T SNP and GD in the dominant genetic model (TT + TC vs. CC). (**a**) Total population; (**b**) Caucasian group.

(Table 3). Five studies assessed the genetic associations of the rs179247 A/G polymorphism with GD^12^,^19^,^20^,^21^,^22^. All of the genetic models, including the allele frequency comparison (G vs. A, OR = 0.76, 95%CI: 0.68–0.85, P = 0.000, I^2^ = 58.2%) and the additive (GA vs. AA, OR = 0.72, 95%CI: 0.65–0.79, P = 0.000, I^2^ = 4.1%; GG vs. AA, OR = 0.53, 95%CI: 0.47–0.60, P = 0.000, I^2^ = 0%), dominant (GG + GA vs. AA, OR = 0.66, 95%CI: 0.61–0.73, P = 0.000, I^2^ = 0%), and recessive models (GG vs. GA + AA, OR = 0.66, 95%CI: 0.59–0.73, P = 0.000, I^2^ = 0%), showed a significant association between rs179247 and a decreased susceptibility to GD for the total group (Fig. 2a). For Caucasians, the allele frequency comparison (G vs. A, OR = 0.77, 95%CI: 0.66–0.91, P = 0.001, I^2^ = 76.5%) and the additive (GA vs. AA, OR = 0.72, 95%CI: 0.65–0.80, P = 0.000, I^2^ = 9.4%; GG vs. AA, OR = 0.53, 95%CI: 0.47–0.61, P = 0.000, I^2^ = 0%), dominant (GG + GA vs. AA, OR = 0.66, 95%CI: 0.60–0.73, P = 0.000, I^2^ = 8.6%), and recessive models (GG vs. GA + AA, OR = 0.66, 95%CI: 0.59–0.75, P = 0.000, I2 = 0%) showed a significant association between rs179247 and a decreased susceptibility to GD (Fig. 2b). Three studies assessed the genetic association between the rs12101255 C/T polymorphism and the risk of developing GD^12^,^19^,^20^. All the genetic models, including the allele frequency comparison (T vs. C, OR = 1.50, 95%CI: 1.41–1.60, P = 0.000, I^2^ = 0%) and the additive (TC vs. CC, OR = 1.51, 95%CI: 1.37–1.67, P = 0.000, I^2^ = 0%; TT vs. CC, OR = 2.24, 95%CI: 1.96–2.56, P = 0.000, I^2^ = 0%), dominant (TT + TC vs. CC, OR = 1.67, 95%CI: 1.53–1.83, P = 0.000, I^2^ = 0%), and recessive models (TT vs. CC + CT, OR = 1.74, 95%CI: 1.55–1.96, P = 0.000, I^2^ = 0%), showed a significant association between rs12101255 and an increased susceptibility to GD for the total population group (Fig. 3a). For Caucasians, the allele frequency comparison (T vs. C, OR = 1.51, 95%CI: 1.41–1.61, P = 0.000, I^2^ = 0%) and the additive (TC vs. CC, OR = 1.52, 95%CI: 1.37–1.68, P = 0.000, I^2^ = 9.8%; TT vs. CC, OR = 2.25, 95%CI: 1.96–2.59, P = 0.000, I^2^ = 0%), dominant (TT + TC vs. CC, OR = 1.67, 95%CI: 1.52–1.84, P = 0.000, I^2^ = 5.6%), and recessive models (TT vs. CC + CT, OR = 1.50, 95%CI: 1.41–1.60, P = 0.000, I^2^ = 0%) showed a significant association between rs12101255 and an increased susceptibility to GD (Fig. 3b). After Bonferroni correction (P > 0.01), the associations were still significant.

Five studies^20^,^22^,^23^,^24^,^25^ assessed the association of the rs179247 A/G and rs2268458 C/T polymorphisms with GO. For the total population, the allele frequency comparison and the additive, dominant and recessive models of rs179247 and rs2268458 showed no association between GO and GD (GD without GO) (Table 3).

For the total population groups, there was significant heterogeneity for the Allelic model of GD rs179247 (I2 = 58.2%, P = 0.026). We also assessed the studies by ethnicity, and the results showed that heterogeneity was still significant for Caucasians (I^2^ = 76.5%, P = 0.005). In his study, Brand did not state the detailed ages and genders, which would be a source of heterogeneity^19^. We excluded this study and found that there was no heterogeneity for both the total group (I^2^ = 0%, P = 0.927) and for Caucasians (I^2^ = 0%, P = 0.903), and the pooled OR was still statistically significant for both the total group (0.72, 0.67–0.77; P = 0.000) and for Caucasians (0.72, 0.67–0.77; P = 0.000). We found some heterogeneity in all of the genetic models for GO rs179247, though these results were not significant (I^2^ ≤ 45.1%). There were three different population groups for GO rs179247, including Caucasian, Chinese and Brazilians that could be the source of heterogeneity.

To explore the effects of the study characteristics on the estimation of effect size, a univariate meta-regression analysis for GD rs179247 was performed. No statistically significant effects were observed, including for the mean age of cases (p = 0.814), the percentage of female cases (p = 0.493) and the sample size of cases (p = 0.953).

### Assessment of sensitivity analysis and potential biases

**Figure 4:**
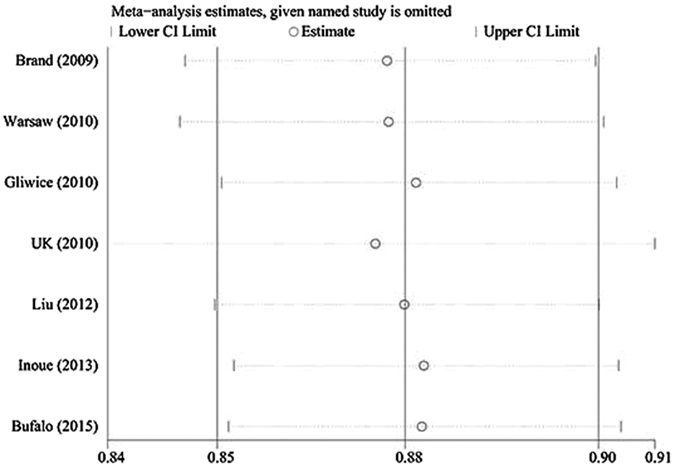
Sensitivity analysis of GD rs179247 in the dominant genetic model (GG + GA vs. AA).

**Figure 5:**
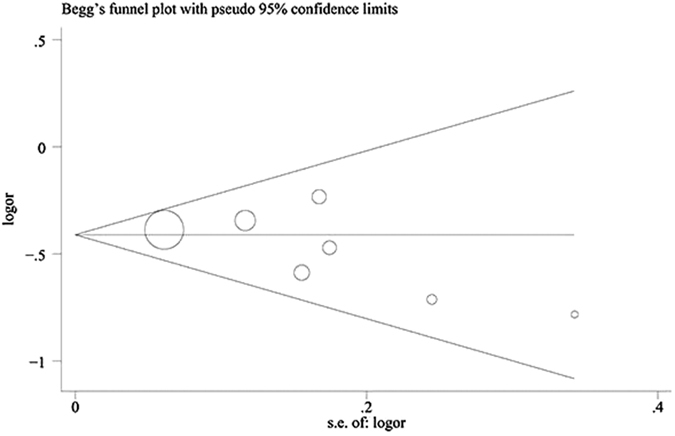
Begg’s funnel plot of GD rs179247 in the dominant genetic model (GG + GA vs. AA).

We performed a sensitivity analysis by excluding each study once in all of the genetic models (Fig. 4). The pooled OR results were stable, which indicated that the results were not influenced by any single study. Begg’s funnel plot (Fig. 5) and Egger’s test (Table 3) were used to evaluate the publication preference of the studies. No publication preference was identified in all of the genetic models by Egger’s test, except the additive model of GD rs12101255 (P = 0.036). The number of relevant studies was small (n = 4), and Egger’s test was not the best method to precisely evaluate the publication partiality. More studies are needed to avoid publication bias. Jurecka compared a subgroup of young GO patients to the entire age group of GO patients for rs179247^23^. We selected the data for the whole age group of GO patients to avoid selection partiality. All of the studies had a ≥7 score from the Newcastle Ottawa Scale (NOS). Therefore, the risk of introducing bias was low, and all of the studies were included.

## Discussion

Graves’ disease is a common autoimmune disease with genetic susceptibility^26^. TSHR is a protein that plays a central role in the pathogenesis of GD and GO. Because of the central role of TSHR in GD genetic studies, the genetic associations between the TSHR gene and GD/GO have been studied for a long time^13^. Many TSHR gene polymorphisms have been discovered. After carefully screening studies with the inclusion criteria, rs179247, rs12101255, and rs2268458 were chosen to evaluate genetic associations with GD/GO susceptibility.

For the total group and the Caucasian group, our results showed that the carriers of rs179247 GG had a 34% less risk of developing GD than the controls. Rs12101255 TT carriers had 26% and 23% more risk of developing GD than the controls in the total group and the Caucasian group, respectively. Allele A of rs179247 and allele T of rs12101255 were more frequent in GD patients. Allele G of rs179247 and allele C of rs12101255 were more common in healthy people.

The location of rs179247 and rs12101255 within the first intron, which is close to the promoter region and the start codon, may influence post-translational processes or gene expression. As the only confirmed disease-specific gene, two mechanisms have been proposed to explain the association of TSHR intron 1 SNPs with the risk of developing GD^13^.

The first mechanism is the alternative splicing process and generation of soluble TSHR (peripheral tolerance). The splicing of exons code for the extracellular domain of the protein is influenced by intronic TSHR polymorphisms. The intronic bases do not exist in the mature mRNA, but they are important in regulating function. The intronic SNPs can influence mRNA splicing and change protein functions. The main transcriptions of TSHR encode 3 different TSHR: the full-length TSHR (flTSHR), ST4 and ST5. ST4 and ST5 have the same 8 exons as flTSHR and an additional ninth exon. Two regions of intron 8 encode the ninth exon so that the introns were retained. In the Brand study that we included in the meta-analysis, the author evaluated flTSHR, ST4 and ST5 expression in 12 thyroid tissue samples^19^. The risk alleles of TSHR SNPs (rs179247AA and rs12101255TT) were associated with a reduced ratio of flTSHR:ST4 and flTSHR:ST5. The increase of ST4 and ST5 resulted in a higher production of the soluble “A” subunit of TSHR in the periphery. This led to the production of thyroid auto-antibodies, which are the cause of GD. The number of thyroid tissue samples was small, and a larger number was needed to answer the question of how intron 1 can influence the alternative splicing of intron 8.

The second mechanism is the modulation of TSHR expression in the thymus (central tolerance). The risk alleles result in a lower TSHR expression in the thymus, which means that less TSHR self-reactive T cells will be deleted. More TSHR-Abs can be produced by the germinal centres of the thyroid-draining lymph nodes. Colobran identified this hypothesis in his study^27^. He found that carriers of the risk allele of rs179247 had significantly less TSHR mRNA transcripts than carriers of the protective allele in the thymic glands of non-autoimmune donors who were homozygous. The level of the TSHR risk allele was lower than that of the protective allele in heterozygous individuals.

TSHR rs179247 and rs2268458 polymorphisms had no association between GO (GD with GO) and GD (GD without GO) for any of the genetic models. In the Jurecka study, which we included in the meta-analysis, the rs179247 TSHR polymorphism had a genetic association with GO for young patients only (age of onset ≤30 years)^23^. For young patients with GO, allele A was statistically more frequent, and homozygous carriers had a significantly lower risk of disease incidence than patients with GG or AG genotypes. There were no differences in elderly patients or in the whole group. The study authors suggested that genetic factors were closely related to the young GO patients and that environmental factors were closely related to the elderly GO patients. No genetic association was found between the rs12101255 TSHR polymorphism with GO in young patients or in the whole group. In the study by Lin and colleagues that we included in the meta-analysis, significant differences were found between GO and healthy controls for the rs179247 and rs12101255 polymorphisms^20^. Compared with healthy controls, allele A of rs179247 was increased in GO patients (P = 0.028, OR = 1.571, 95%CI = 1.047–2.357). Allele T of rs12101255 was also significantly higher in both GO and GD patients compared to healthy controls. In the study by Yin and colleagues that we included in the meta-analysis, rs2268458 was also found to be genetically associated with GD, but not with GO^24^.

TSHR is the auto antigen for both GD and GO. The pathogenesis of GD/GO is complex and multifactorial with many environmental and genetic factors. Environmental factors, such as smoking and old age, are considered risk factors for GO. However, the genetic associations of TSHR between GD and GO are still unclear. Compared with GD (GD without GO) patients, no SNPs were associated with GO (GD with GO) in our meta-analysis even though these SNPs were positively associated with GD. Because 25–50% of GD patients have a clinical manifestation of GO, more studies are needed to identify aetiological factors besides the gene.

As far as we know, our study is the first meta-analysis to evaluate the genetic association of TSHR polymorphisms with GO. We searched all the available data with no language limits. Studies of high quality were selected with inclusion and exclusion criteria. We also performed sub-group analysis, meta-regression analysis and sensitivity analysis to test the reliability of the statistical results. Hardly any publication unfairness was identified by either Begg’s funnel plot or Egger’s regression test. As such, the data analysis was stable and reliable. The central and tissue-specific role of TSHR gene was further confirmed with GD/GO in our meta-analysis.

Some limitations of the genetic studies in GD/GO were revealed. First, Caucasian and Asian people were the primary study population groups. Other ethnic information was limited. More ethnic data are needed for further studies. Second, the number of TSHR genetic studies in GO was small. Therefore, more studies are needed to identify the genetic associations of TSHR with GO. Third, genetic studies in GD/GO should collect more evidence of the environmental, hormonal and antigenic factors and the interactions of these factors with genetic factors. These data would strengthen our understanding of the multi-factorial pathogenesis of GD/GO.

In summary, we identified TSHR rs179247 and rs12101255 as genetically associated with GD. TSHR rs12101255 and rs2268458 polymorphisms had no genetic association between GD and GO. Future studies with bigger sample sizes and different ethnic population groups are needed to confirm the genetic association of TSHR gene polymorphisms with GD/GO. In addition, TSHR genetic studies should investigate gene-environment as well as gene-gene interactions. This approach would lead to a more precise understanding of the genetic association of TSHR gene polymorphisms with GD/GO.

## Methods

### Search strategy

We searched Pub Med and EMBASE for case-control studies published up to December 2015 that studied the genetic association of TSHR gene polymorphisms with GD/GO. The search strategy was based on a combination of “(thyroid stimulating hormone receptor gene OR TSHR gene) AND (gene OR variants OR polymorphism OR alleles OR mutation) AND (Graves’ disease OR Graves’ ophthalmopathy OR thyroid-associated ophthalmopathy)”.

We also manually scanned the references in articles and reviews to include all potentially relevant articles with no language limitations^28^.

### Inclusion and Exclusion Criteria

The inclusion criteria were as follows: (a) studied the association of TSHR gene polymorphisms with the GD/GO; (b) was a case-control study; (c) had sufficient published data for estimating an odds ratio (OR) with a 95% confidence interval (CI); and (d) diagnosis of GD was based on standard clinical criteria, including increased serum levels of free thyroxine (FT4) and triiodothyronine (T3), decreased TSH, positive TRAb values, presence of a diffuse goitre as detected by ultrasonography, and increased thyroidal uptake of pertechnetate. The eye examinations included proptosis measurement; measurement of eyelid width; assessment of muscular function; assessment of corneal status; examination of the fundus oculi; and measurements of visual acuity. The exclusion criteria were as follows: animal studies, case reports, reviews, abstracts, conference proceedings, editorials or studies with incomplete data.

### Literature Review and Data Extraction

Two investigators (H.B.X. and M.X.W.) screened and reviewed each article and completed the data extraction independently. Disagreements were resolved by group discussion with a third investigator (X.Y.Z.). To study the genetic associations of the TSHR gene polymorphisms with GD/GO, we selected the most strongly associated SNPs to analyse (rs179247, rs12101255 and rs2268458). Data were extracted by two investigators (H.B.X. and M.X.W.), and the data included the first author, publication year, location, population ethnicity, definition and sample of the cases and controls, gender and mean age of cases, and the DNA extraction and genotype results. The Hardy-Weinberg equilibrium (HWE) was used to test the genetic equilibrium^29^. The study authors were contacted for supplemental data.

### Statistical analysis

Each SNP was used for meta-analysis if there were 2 or more studies. Different genetic models were used to assess the genetic association, including the allelic (B vs. A), dominant (AB + BB vs. AA), recessive (BB vs. AA + AB) and codominant (homozygous: BB vs. AA; heterozygous: AB vs. AA) models^29^. A is a wild type gene, and B is a mutant gene. The strength of association was assessed using the summary OR with a 95% CI for each SNP^30^. The heterogeneity was evaluated with a Q- statistic and the I^2^ value. Statistically, a larger amount was considered when P < 0.05. If the P value was ≥0.05 or the I^2^ value <50%, a fixed-effect model was used, and then a random-effect model was adopted^31^. A Z-test was used to test the significance of the pooled OR, and P < 0.05 was considered to be statistically significant^32^. Studies were analysed by ethnicity to minimize possible heterogeneity. Sensitivity analyses were completed to explore the reasons for heterogeneity and to evaluate the stability of the results by sequentially excluding each study. Begg’s funnel plot and Egger’s test were used to evaluate publication bias. If the P value of Egger’s test was <0.05, a statistically significant publication bias was considered. Because 5 genetic models were used, P < 0.01 (0.05/5) was considered to be statistically significant for association^29^. The HWE was tested if it was not stated in the original study. The Newcastle Ottawa Scale (NOS) was used to test the study quality by two independent reviewers (accessed via http://www.ohri.ca/programs/clinical epidemiology/oxford.asp)^29^. Three dimensions were carefully checked: (1) selection, 0–4; (2) comparability, 0–2; and (3) exposure, 0–3. The study quality was considered to be good with a score ≥7. Any disagreement was resolved by a third author.

All calculations were performed using Stata 12.1 (StataCorp, College Station, Texas).

## Additional Information

**How to cite this article**: Xiong, H. *et al*. Genetic associations of the thyroid stimulating hormone receptor gene with Graves diseases and Graves ophthalmopathy: A meta-analysis. *Sci. Rep.* **6**, 30356; doi: 10.1038/srep30356 (2016).

## Figures and Tables

**Table 1:** Main characteristics of the GD/GO studies included in the meta-analysis.

No.	Study (year)	Country	Ethnicity	TSHR SNP	Definition			Case females n (%)	Case mean age(year)	HWE*	NOS
					cases	controls	Sample size				
1	Brand (2009)	UK	Caucasian	rs179247A/G rs12101255C/T	GD	Health	768/768	NA	NA	YES	7
2	Ploski (2010)	Warsaw Gliwice UK	Caucasian	rs179247A/G rs12101255C/T	GD	Health	558/520 196/1982504/2784	448 (80.3) 161 (82.1) 2047 (82.8)	39.6 44.2 43.1	YES	8
3	Liu (2012)	China	Chinese	rs179247A/G rsl2101255C/T	GD	Health	404/242	290(71.79)	34.21	YES	8
4	Inoue (2013)	Japan	Japanese	rs179247 A/G	GD	Health	112/56	95(84.8)	34.3	YES	8
5	Bufalo (2015)	Brazil	Brazilian	rs179247 A/G	GD	Health	279/296	231(82.8)	39.8	YES	8
6	Yin (2008)	USA	Caucasian	rs2268458 T/C	GO	GD without GO	120/80	120(100)	48	YES	8
7	Yin (2012)	USA	Caucasian	rs2268458 T/C	GO	GD without GO	256/90	198(77.3)	52	YES	8
8	Liu (2012)	China	Chinese	rs179247A/G	GO	GD without GO	101/303	n.a.	34.2	YES	7
9	Jurecka (2014)	Poland	Caucasian	rs179247A/G	GO	GD without GO	283/316	n.a.	40.3	YES	7
10	Bufalo (2015)	Brazil	Brazilian	rs179247 A/G	GO	GD without GO	144/135	111(77.1)	40.1	YES	8

HWE, Hardy-Weinberg equilibrium; TSHR, thyroid-stimulating hormone receptor; n.a., not available; GD, Graves’ disease; GO, Graves’ ophthalmopathy; SNP, single nucleotide polymorphism; NOS*, Newcastle Ottawa Scale. The genetic equilibrium of the TSHR gene for the control group of each study was evaluated by testing for HWE using chi-square analyses. Disequilibrium was defined as P < 0.05.

**Table 2:** Genotype frequencies of the TSHR SNPs in the studies included in the meta-analysis.

SNP	Study	Ethnicity	Case			Control			Genotyping method
			1*	2*	3*	1*	2*	3*	
GD rs179247A/G	Brand (2009)	Caucasian	279	359	100	182	322	100	TaqMan
	Ploski (2010)	Caucasian	139	270	149	81	259	180	TaqMan
		Caucasian	58	84	54	34	98	67	
		Caucasian	879	1110	351	737	1243	561	
	Liu (2012)	Chinese	230	140	24	120	88	20	MALDI-TOF-MS
	Inoue (2013)	Japanese	57	44	11	18	33	5	PCRRFLP
	Bufalo (2015)	Brazilian	117	138	24	92	154	50	TaqMan
GD rs12101255C/T	Brand (2009)	Caucasian	197	345	150	268	295	89	TaqMan
	Ploski (2010)	Caucasian	238	245	75	273	212	35	TaqMan
		Caucasian	70	94	32	110	71	17	
		Caucasian	687	1136	482	1046	1148	338	
	Liu (2012)	Chinese	38	179	187	35	119	86	MALDI-TOF-MS
GO rs2268458 T/C	Yin (2008)	Caucasian	67	43	10	38	32	10	PCR-RFLP
	Yin (2012)	Caucasian	139	99	18	44	36	10	PCR-RFLP
GO rs179247A/G	Liu (2012)	Chinese	62	33	3	168	107	21	MALDI-TOF-MS
	Jurecka (2014)	Caucasian	80	126	77	91	148	77	PCR-RFLP
	Bufalo (2015)	Brazilian	54	78	12	63	60	12	TaqMan

(1*, Homozygous wild type; 2*, Heterozygous variant; 3*, Homozygous variant).

**Table 3:** Summary of the pooled odds ratios for the association of the TSHR gene and GD/GO in the meta-analysis.

SNP	Ethnicity	Genetic model	Total allele or genotype counts		OR (95% CI)	P	Heterogeneity test		Egger’s Test(P)
			Case	Control			I^2^	P	
GD rs179247	Total	G vs. A	9354	8888	0.76(0.68–0.85)	0.000	58.2	0.026	0.996
		GA vs. AA	3904	3461	0.72(0.65–0.79)	0.000	4.1	0.395	0.095
		GG vs. AA	2472	2247	0.53(0.47–0.60)	0.000	0	0.700	0.867
		GG + GA vs. AA	4617	4444	0.66(0.61–0.73)	0.000	0	0.456	0.245
		GG vs. GA + AA	4617	4444	0.66(0.59–0.73)	0.000	0	0.537	0.327
	Caucasian	G vs. A	4680	5082	0.77(0.66–0.91)	0.001	76.5	0.005	0.798
		GA vs. AA	1989	1980	0.72(0.65–0.80)	0.000	9.4	0.346	0.084
		GG vs. AA	1230	1298	0.53(0.47–0.61)	0.000	0	0.586	0.378
		GG + GA vs. AA	2340	2541	0.66(0.60–0.73)	0.000	8.6	0.350	0.648
		GG vs. GA + AA	2340	2541	0.66(0.59–0.75)	0.000	0	0.497	0.050
GD rs12101255	Total	T vs. C	8310	8284	1,50(1.41–1.60)	0.000	0	0,642	0.423
		TC vs. CC	3229	3577	1.51(1.37–1.67)	0.000	0	0.487	0.623
		TT vs. CC	2156	2297	2.24(1.96–2.56)	0.000	0	0.822	0.265
		TT + TC vs. CC	4155	4142	1.67(1.53–1.83)	0.000	0	0.528	0.501
		TT vs. CC + CT	4155	4142	1.74(1.55–1.96)	0.000	0	0.766	0.345
	Caucasian	T vs. C	7502	7804	1.51(1.41–1.61)	0.000	0	0.530	0.248
		TC vs. CC	3012	3423	1.52(1.37–1.68)	0.000	9.8	0.344	0.480
		TT vs. CC	1931	2176	2.25(1.96–2.59)	0.000	0	0.801	0.036
		TT + TC vs. CC	3751	3902	1.67(1.52–1.84)	0.000	5.6	0.365	0.455
		TT vs. CC + CT	3751	3902	1,50(1.41–1.60)	0.000	0	0,642	0.139
GO rs179247	Total	G vs. A	1050	1494	1.03(0.86–1.22)	0.775	45.1	0.162	0.744
		GA vs. AA	433	637	1.05(0.81–1.35)	0.717	36.2	0.209	0.722
		GG vs. AA	288	432	1.01(0.70–1.45)	0.966	25.2	0.263	0.469
		GG + GA vs. AA	525	747	1.04(0.81–1.32)	0.768	44.5	0.165	0.875
		GG vs. GA + AA	525	747	1.02(0.74–1.41)	0.885	22.3	0.276	0.173
GO rs2268458	Total	C vs. T	752	340	0.77(0.58–1.02)	0.07	0	0.811	n.a.
		CT vs. TT	348	150	0.82(0.56–1.22)	0.33	0	0.742	n.a.
		CC vs. TT	234	102	0.57(0.30–1.07)	0.081	0	0.994	n.a.
		CT + CC vs. TT	376	170	0.77(0.53–1.11)	0.155	0	0.756	n.a.
		CC vs. TT + TC	376	170	0.62(0.34–1.14)	0.124	0	0.936	n.a.

n.a.: not available. Publication bias could not be evaluated because a minimum of 3 studies was required.

## References

[b1] Burch H. B. & Cooper D. S. Management of Graves Disease: A Review. JAMA 314, 2544–2554 (2015). 26670972 10.1001/jama.2015.16535

[b2] Hollowell J. G. . Serum TSH, T(4), and thyroid antibodies in the United States population (1988 to 1994): National Health and Nutrition Examination Survey (NHANES III). J Clin Endocrinol Metab 87, 489–499 (2002). 11836274 10.1210/jcem.87.2.8182

[b3] Bartalena L. & Fatourechi V. Extrathyroidal manifestations of Graves’ disease: a 2014 update. J Endocrinol Invest 37, 691–700 (2014). 24913238 10.1007/s40618-014-0097-2

[b4] Piantanida E., Tanda M. L., Lai A., Sassi L. & Bartalena L. Prevalence and natural history of Graves’ orbitopathy in the XXI century. J Endocrinol Invest 36, 444–449 (2013). 23587873 10.3275/8937

[b5] Selmi-Ruby S. . The targeted inactivation of TRbeta gene in thyroid follicular cells suggests a new mechanism of regulation of thyroid hormone production. Endocrinology 155, 635–646 (2014). 24265449 10.1210/en.2013-1435

[b6] Davies T. F., Ando T., Lin R. Y., Tomer Y. & Latif R. Thyrotropin receptor-associated diseases: from adenomata to Graves disease. J Clin Invest 115, 1972–1983 (2005). 16075037 10.1172/JCI26031 PMC1180562

[b7] Smith B. R., Sanders J. & Furmaniak J. TSH receptor antibodies. Thyroid 17, 923–938 (2007). 17900238 10.1089/thy.2007.0239

[b8] Dik W. A., Virakul S. & van Steensel L. Current perspectives on the role of orbital fibroblasts in the pathogenesis of Graves’ ophthalmopathy. Exp Eye Res 142, 83–91 (2016). 26675405 10.1016/j.exer.2015.02.007

[b9] Iyer S. & Bahn R. Immunopathogenesis of Graves’ ophthalmopathy: the role of the TSH receptor. Best Pract Res Clin Endocrinol Metab 26, 281–289 (2012). 22632365 10.1016/j.beem.2011.10.003 PMC3361679

[b10] Eckstein A. K. . Thyrotropin receptor autoantibodies are independent risk factors for Graves’ ophthalmopathy and help to predict severity and outcome of the disease. J Clin Endocrinol Metab 91, 3464–3470 (2006). 16835285 10.1210/jc.2005-2813

[b11] Simmonds M. J. GWAS in autoimmune thyroid disease: redefining our understanding of pathogenesis. Nat Rev Endocrinol 9, 277–287 (2013). 23529038 10.1038/nrendo.2013.56

[b12] Ploski R. . Thyroid stimulating hormone receptor (TSHR) intron 1 variants are major risk factors for Graves’ disease in three European Caucasian cohorts. PLoS One 5, e15512 (2010). 21124799 10.1371/journal.pone.0015512 PMC2991361

[b13] Pujol-Borrell R., Gimenez-Barcons M., Marin-Sanchez A. & Colobran R. Genetics of Graves’ Disease: Special Focus on the Role of TSHR Gene. Horm Metab Res 47, 753–766 (2015). 26361261 10.1055/s-0035-1559646

[b14] Chu X. . A genome-wide association study identifies two new risk loci for Graves’ disease. Nat Genet 43, 897–901 (2011). 21841780 10.1038/ng.898

[b15] Wellcome Trust Case Control C. . Association scan of 14,500 nonsynonymous SNPs in four diseases identifies autoimmunity variants. Nat Genet 39, 1329–1337 (2007). 17952073 10.1038/ng.2007.17 PMC2680141

[b16] Allahabadia A. . Lack of association between polymorphism of the thyrotropin receptor gene and Graves’ disease in United Kingdom and Hong Kong Chinese patients: case control and family-based studies. Thyroid 8, 777–780 (1998). 9777748 10.1089/thy.1998.8.777

[b17] Ban Y., Greenberg D. A., Concepcion E. S. & Tomer Y. A germline single nucleotide polymorphism at the intracellular domain of the human thyrotropin receptor does not have a major effect on the development of Graves’ disease. Thyroid 12, 1079–1083 (2002). 12593721 10.1089/105072502321085171

[b18] Khalilzadeh O., Noshad S., Rashidi A. & Amirzargar A. Graves’ ophthalmopathy: a review of immunogenetics. Curr Genomics 12, 564–575 (2011). 22654556 10.2174/138920211798120844 PMC3271309

[b19] Brand O. J. . Association of the thyroid stimulating hormone receptor gene (TSHR) with Graves’ disease. Hum Mol Genet 18, 1704–1713 (2009). 19244275 10.1093/hmg/ddp087

[b20] Liu L. . Association between thyroid stimulating hormone receptor gene intron polymorphisms and autoimmune thyroid disease in a Chinese Han population. Endocr J 59, 717–723 (2012). 22673349 10.1507/endocrj.ej12-0024

[b21] Inoue N., Watanabe M., Katsumata Y., Hidaka Y. & Iwatani Y. Different genotypes of a functional polymorphism of the TSHR gene are associated with the development and severity of Graves’ and Hashimoto’s diseases. Tissue Antigens 82, 288–290 (2013). 23962080 10.1111/tan.12190

[b22] Bufalo N. E. . TSHR intronic polymorphisms (rs179247 and rs12885526) and their role in the susceptibility of the Brazilian population to Graves’ disease and Graves’ ophthalmopathy. J Endocrinol Invest 38, 555–561 (2015). 25543543 10.1007/s40618-014-0228-9

[b23] Jurecka-Lubieniecka B. . Association between polymorphisms in the TSHR gene and Graves’ orbitopathy. PLoS One 9, e102653 (2014). 25061884 10.1371/journal.pone.0102653 PMC4111286

[b24] Yin X., Latif R., Bahn R., Tomer Y. & Davies T. F. Influence of the TSH receptor gene on susceptibility to Graves’ disease and Graves’ ophthalmopathy. Thyroid 18, 1201–1206 (2008). 18925838 10.1089/thy.2008.0098 PMC2857451

[b25] Yin X., Latif R., Bahn R. & Davies T. F. Genetic profiling in Graves’ disease: further evidence for lack of a distinct genetic contribution to Graves’ ophthalmopathy. Thyroid 22, 730–736 (2012). 22663548 10.1089/thy.2012.0007 PMC3387758

[b26] Fang Y. . Genetic association of Fc receptor-like glycoprotein with susceptibility to Graves’ disease in a Chinese Han population. Immunobiology 221, 56–62 (2016). 26321232 10.1016/j.imbio.2015.08.002

[b27] Colobran R. . Association of an SNP with intrathymic transcription of TSHR and Graves’ disease: a role for defective thymic tolerance. Hum Mol Genet 20, 3415–3423 (2011). 21642385 10.1093/hmg/ddr247

[b28] Au A. . The Influence of OLR1 and PCSK9 Gene Polymorphisms on Ischemic Stroke: Evidence from a Meta-Analysis. Sci Rep 5, 18224 (2015). 26666837 10.1038/srep18224 PMC4678327

[b29] Wong K. H. . Genetic Associations of Interleukin-related Genes with Graves’ Ophthalmopathy: a Systematic Review and Meta-analysis. Sci Rep 5, 16672 (2015). 26578206 10.1038/srep16672 PMC4649612

[b30] Lu Y. . Genetic association of RIT2 rs12456492 polymorphism and Parkinson’s disease susceptibility in Asian populations: a meta-analysis. Sci Rep 5, 13805 (2015). 26334395 10.1038/srep13805 PMC4558715

[b31] Ma L. . Association of PEDF polymorphisms with age-related macular degeneration and polypoidal choroidal vasculopathy: a systematic review and meta-analysis. Sci Rep 5, 9497 (2015). 25820866 10.1038/srep09497 PMC4377572

[b32] Liu K. . Ethnic differences in the association of SERPING1 with age-related macular degeneration and polypoidal choroidal vasculopathy. Sci Rep 5, 9424 (2015). 25800435 10.1038/srep09424 PMC4371106

